# Inferring genetic structure when there is little: population genetics versus genomics of the threatened bat *Miniopterus schreibersii* across Europe

**DOI:** 10.1038/s41598-023-27988-4

**Published:** 2023-01-27

**Authors:** Christophe Dufresnes, Ludovic Dutoit, Alan Brelsford, Fardo Goldstein-Witsenburg, Laura Clément, Adria López-Baucells, Jorge Palmeirim, Igor Pavlinić, Dino Scaravelli, Martin Ševčík, Philippe Christe, Jérôme Goudet

**Affiliations:** 1 Laboratory for Amphibian Systematic and Evolutionary Research, College of Biology and the Environment, Nanjing Forestry University, Nanjing, People’s Republic of China; 2 Department of Ecology and Evolution, University of Lausanne, 1015 Lausanne, Switzerland; 3 Department of Zoology, University of Otago, Dunedin, New Zealand; 4 Department of Evolution, Ecology, and Organismal Biology, University of California Riverside, Riverside, CA USA; 5 Bat Research Area, Granollers Museum of Natural Sciences, Carrer Palaudaries 102, 08402 Granollers, Spain; 6 Department of Animal Biology, Centre for Ecology, Evolution and Environmental Change – cE3c, University of Lisbon, 1749-016 Lisbon, Portugal; 7 Department of Zoology, Croatian Natural History Museum, Demetrova 1, 10000 Zagreb, Croatia; 8 Department of Biological, Geological, and Environmental Sciences, University of Bologna, Via Selmi 3, 40126 Bologna, Italy; 9 Department of Zoology, Faculty of Science, Charles University in Prague, Viničná 7, 128 44 Prague 2, Czech Republic

**Keywords:** Ecology, Evolution, Genetics, Zoology

## Abstract

Despite their paramount importance in molecular ecology and conservation, genetic diversity and structure remain challenging to quantify with traditional genotyping methods. Next-generation sequencing holds great promises, but this has not been properly tested in highly mobile species. In this article, we compared microsatellite and RAD-sequencing (RAD-seq) analyses to investigate population structure in the declining bent-winged bat (*Miniopterus schreibersii*) across Europe. Both markers retrieved general patterns of weak range-wide differentiation, little sex-biased dispersal, and strong isolation by distance that associated with significant genetic structure between the three Mediterranean Peninsulas, which could have acted as glacial refugia. Microsatellites proved uninformative in individual-based analyses, but the resolution offered by genomic SNPs illuminated on regional substructures within several countries, with colonies sharing migrators of distinct ancestry without admixture. This finding is consistent with a marked philopatry and spatial partitioning between mating and rearing grounds in the species, which was suspected from marked-recaptured data. Our study advocates that genomic data are necessary to properly unveil the genetic footprints left by biogeographic processes and social organization in long-distant flyers, which are otherwise rapidly blurred by their high levels of gene flow.

## Introduction

A comprehensive picture of the genetic structure of populations is instrumental to any ecological, evolutionary and conservation research^1^. Patterns of genetic variation, differentiation and admixture inform on the history of populations and thus help understanding the ecological and geographic processes shaping biodiversity in space and time^2–4^. Molecular assessments also offer a swift alternative to traditional monitoring techniques (e.g., mark-recapture), especially when it comes to measuring individual movements, population fragmentation and other key parameters relevant for conservation such as inbreeding and local adaptation^5–7^. As such, genetic surveys have become an essential part of the management of threatened species^8,9^, by assisting the design of conservation strategies to enhance population connectivity^10^, restore bottlenecked populations^11^, and plan translocation/reintroduction efforts in respect to evolutionary units^12^.

In many instances, however, inferring genetic structure is far from trivial. Patterns of population differentiation may be difficult to detect for mobile organisms, as populations are homogenized by recurrent episodes of gene flow^13,14^. The issue is also characteristic of genetically impoverished species that experienced range-wide demographic changes, such as refugial bottlenecks and post-glacial expansions, and for which the remaining diversity might be too low to quantify population connectivity^4^. Moreover, at times in the life cycle, momentary gatherings of individuals from various geographic origins might also blur the appreciations of genetic diversity and between-population differentiation, as seasonal migration confound with dispersal^15^. In such cases, a high genetic resolution can offer insights into individual ancestries that distinguish seasonal migrators *vs* dispersers.

In theory, the accuracy of population genetic analyses should depend on the number of loci used in respect to their standing variation. Microsatellites combine the advantages of evolving neutrally^16,17^ and experiencing high mutation rates that generate myriads of alleles. They accordingly remain popular markers to estimate gene flow and diversity among recently diverged populations ever since the late 1990s^18,19^. Also highly polymorphic, mitochondrial DNA (mtDNA) is more straightforward and cheaper to analyze, notably across evolutionary distant populations, and has accordingly been preferred by researchers interested in range-wide phylogeographic structure^20^. However, mtDNA faces inherent limitations with respect to its clonal nature and maternal mode of inheritance, which makes it more sensitive to selection^21^ and can bias the reconstruction of the demographic history of populations, especially when dispersal is sex-biased^22–24^. In recent years, molecular ecologists benefit from high-throughput sequencing technology to access unprecedented numbers of loci, as prepared by genomic libraries^25,26^. In particular, restriction-site associated DNA sequencing (RAD-seq^27^; double-digest ddRAD-seq^28^) have become a popular method to genotype hundreds to thousands single nucleotide polymorphisms (SNPs) across conspecific or interspecific populations for multiple applications, such as high-resolution phylogeography^29^ and population genetic studies of weakly structured species^4,28^. The number of alleles of a single microsatellite locus is much higher than that of SNPs, but microsatellite analyses rarely include more than 10–20 loci due to practical constraints. By generating several hundred times more loci, RAD-seq thus largely compensates for the lower per-locus diversity of SNPs to provide more powerful population genetic inferences, notably in situations when gene exchange is hard to apprehend. However, such improvement has not been satisfactorily quantified, since the few comparative studies have focused on species with relatively strong levels of population differentiation, where structure is already detected efficiently by microsatellites (reviewed by^30^).

In this study, we assessed how much RAD-seq can clarify subtle patterns of population structure compared to microsatellites in one of Earth’s widespread mammals, the bent-winged bat *Miniopterus schreibersii*. This species was once considered to extend from Western Europe to Australasia, but taxonomic changes have now restricted its range to the Western Palearctic, where it is mostly found around the Mediterranean Basin (Southern Europe, Asia Minor and North-Africa). Bats in general are models of choice to gauge the resolution of genetic markers. Their high mobility usually combined with female philopatry and sex-biased dispersal^31^ implies pervasive gene flow and cyto-nuclear discordances^32^, thus inducing substantial difficulties to retrieve clear signals of population structure from traditionally used loci. In this respect, *M. schreibersii* is a long-distance traveler^33^ that conducts fixed seasonal migrations between hibernacula, maternity roosts and mating sites by movements of 40–100 km on average, but up to several hundred kilometers^34,35^. Nevertheless, young or adult dispersal overall remain rare in this species, so regional rather than long-distance movements have been argued to contribute most of the genetic exchanges^36^. As a consequence, the composition of nursery and hibernation sites were shown to differ^37,38^, so individuals of independent origins could temporarily share the same sites.

Despite substantial sampling and genotyping efforts, previous studies reported little genetic structure across the vast range of the species. Mitochondrial analyses found a single mitochondrial clade, with closely related haplotypes shared all over the Mediterranean Basin^39^, and microsatellite analyses did not detect meaningful geographic patterns of nuclear differentiation^40^. Both results were interpreted as the consequence of the biogeographic history of the species, namely the massive recolonization of Europe after the last ice age from a single refugium, presumably in Anatolia^39,40^. While this hypothesis has merit, such strong genetic homogeneity at the continental scale may equally reflect the lack of informativeness of conventional genetic markers to detect shallow levels of population differentiation, for instance, if refugial structure had been compromised by high levels of post-glacial gene flow in this mobile species. Likewise, these may overlook subtle genetic signals caused by the social organization of the species. Precise knowledge of the genetic diversity and genetic structure would also be relevant to guide conservation planning. Despite its wide distribution, *M. schreibersii* is facing dramatic declines in Europe since the mid-twentieth century, even being extirpated from several countries in recent years, to the point that it is presently considered Vulnerable by the IUCN Red List (VU A2c)^41^.

How biogeographic history, social organization and recent declines have interacted with philopatry, regional dispersal and long-distance migration to shape the genetic structure of *M. schreibersii* thus remains an open question, especially given the (lack of) geographic patterns retrieved from previous studies. Genomic analyses would thus be timely, and in turn offer an opportunity to assess the molecular resolution required to detect genetic structure when there is little. To this end, here we analyzed regional sets of population and individual samples across the western and Central European ranges of *M. schreibersii* with microsatellite loci and SNPs obtained from ddRAD-seq. The outcomes of both approaches were directly compared by quantitative (fixation indices, heterozygosity, pairwise differentiation) and qualitative (clustering assignment) measures. While RAD-seq is generally expected to provide more accurate estimates, it should be particularly more informative than microsatellites to detect phylogeographic and regional structure, even if populations frequently exchange genes and migratory individuals.

## Methods

### DNA sampling and extraction

A total of 196 adult individuals of *M. schreibersii* were sampled from 19 localities covering seven European regions during spring and summer 2010, 2011 and 2012 (Table 1). Bats were caught by mist nets or harp traps at the entrance of roosting caves, either upon emergence or by entering the roost during the day. For each individual, blood was sampled by puncturing the uropatagial vein with a 0.5 mm gauge needle (Neolus ®). 10–30 μl volumes were collected with heparinized capillaries (Assistent ®), smeared on filter paper, and dried in an envelope for field storage at room temperature. A homeostatic cotton was applied on the wound until the bleeding has stopped. In addition, a 1 mm Ø biopsy punch was applied on the wing membrane (Stiefel ®) and the tissue was stored in 96% ethanol. Bats were released immediately at the site of capture. All experimental protocols (animal capture and tissue sampling) were approved by relevant committees, namely the Service Vétérinaire du Canton de Vaud for Switzerland (2322), the Ministry of Environment of the Slovak Republic for Slovakia (2598/715/03-5.1 pil), the Departament d’Agricultura, Ramaderia, Pesca, Alimentació i Medi Natural de la Generalitat de Catalunya for Spain (SF/379), the Direction for Nature Protection of the Ministry of Culture for Croatia (URBROJ 532-08-01-04/3-10-02), the Minister dell’Ambiante for Italy (7588), the DREAL of (former) region Aquitaine for France, and the Instituto para a Conservação da Natureza e Biodiversidade for Portugal. All methods were carried out in accordance with relevant guidelines and regulations.

**Table 1:** Sample origins, sizes, time of collection, and population genetic estimates inferred from 11 microsatellites and 4994 SNPs obtained by RAD-seq.

	X	Y	n	Microsatellites					RAD-seq SNPs				
				H_O_	H_S_	F_IS_	A_R_	Beta	H_O_	H_S_	F_IS_	A_R_	Beta
**Portugal (05.2011)**													
1	− 8.18	37.24	9	0.52	0.46	− 0.12	2.66	− 0.01	0.12	0.13	0.03	1.13	0.07
2	− 7.66	37.33	10	0.47	0.45	− 0.06	2.58	0.01	0.13	0.13	0.02	1.13	0.03
3	− 8.67	37.77	15	0.44	0.46	0.05	2.67	− 0.01	0.13	0.13	0.02	1.14	0.02
4	− 8.42	39.73	10	0.37	0.38	0.02	2.40	0.17	0.12	0.12	− 0.01	1.12	0.15
**Spain (05.2011)**													
5	2.02	41.33	10	0.39	0.41	0.02	2.43	0.10	0.13	0.13	0.01	1.13	0.08
6	2.73	41.65	9	0.47	0.43	− 0.12	2.59	0.06	0.12	0.13	0.01	1.13	0.08
7	2.51	42.04	10	0.40	0.44	0.09	2.53	0.03	0.13	0.13	0.01	1.13	0.05
8	2.87	42.00	13	0.43	0.45	0.09	2.63	0.02	0.12	0.13	0.04	1.13	0.08
**France (09.2011)**													
9	0.35	45.04	5	0.35	0.38	0.07	2.24	0.18	0.09	0.11	0.10	1.11	0.24
**Switzerland (04–10.2010)**													
10	6.52	46.79	7	0.49	0.43	− 0.12	2.49	0.05	0.12	0.12	0.02	1.12	0.12
**Italy (08.2012)**													
11	10.73	43.40	14	0.37	0.42	0.12	2.54	0.08	0.12	0.13	0.05	1.13	0.10
12	11.67	44.26	12	0.45	0.48	0.06	2.78	− 0.05	0.11	0.12	0.06	1.12	0.11
13	12.44	43.94	15	0.36	0.40	0.08	2.56	0.12	0.12	0.12	0.02	1.12	0.11
**Croatia (05.2011)**													
14	15.66	44.14	6	0.40	0.41	0.01	2.40	0.11	0.13	0.13	− 0.01	1.13	0.07
15	16.37	43.99	11	0.43	0.44	0.04	2.63	0.03	0.13	0.14	0.03	1.14	− 0.01
16	16.10	43.78	13	0.42	0.44	0.06	2.77	0.04	0.13	0.14	0.03	1.14	0.02
17	16.67	43.79	9	0.41	0.44	0.03	2.68	0.03	0.13	0.13	0.00	1.13	0.04
**Slovakia (08.2012)**													
18	20.18	48.62	9	0.41	0.43	0.03	2.48	0.05	0.10	0.13	0.11	1.12	0.12
19	20.95	48.62	9	0.41	0.44	0.05	2.61	0.04	0.12	0.14	0.08	1.13	0.03

All sites are nursing sites, except Switzerland, which is also used as a transit site during fall, and sometimes hibernating sites.

H_O_, observed heterozygosity; H_S_, expected heterozygosity; F_IS_, inbreeding coefficient; A_R_, allelic richness; Beta, population specific F_ST_.

DNA was extracted using the DNeasy Blood & Tissue Kit (Qiagen ®) following the manufacturer’s instructions except for the following particularities. Blood-soaked paper fragments (~ 10 mm in diameter) were cut in small pieces with sterile scissors, and incubated in 360 µL of ATL buffer at 90 °C for 15 min. After cooling, 40 µL of Proteinase K was added to each sample and left for overnight digestion at 56 °C. Elution was performed in 55 µL volumes and by incubating 15 min at 37 °C prior to centrifugation. This step was repeated a second time to maximize DNA yields. For a subset of individuals, additional DNA was extracted from the wing biopsy after rinsing the ethanol-preserved tissues by pure MilliQ water soaking (Millipore ®).

### Microsatellites genotyping

Individuals were genotyped at 11 polymorphic microsatellite loci amplified in four multiplex PCRs^42,43^. Loci, primers, and amplification protocols are detailed in^44^. Amplicons were quality-checked on an agarose gel, typed on an ABI Prism 3100 genetic analyser (Applied Biosystems ®) and size-scored with GeneMapper (Applied Biosystems). Finally, we checked for large allelic drop out, stuttering and null alleles using Micro-Checker v2.2.3^45^.

### ddRAD-seq

For genomic library preparation, extracted samples were first concentrated using a chloropane precipitation. We then prepared a ddRAD library modified from^46^, as detailed in Supplementary File S1. Three separate libraries were multiplexed and sequenced on two lanes of an Illumina HiSeq2000 (Lausanne Genomic Technologies Facility, Switzerland).

Sequences with low signal-to-noise ratio (< 0.6) were discarded using Illumina’s Chastity filter. In the absence of a reference genome, demultiplexing, stacking and cataloguing of reads was performed with the de-novo pipeline of STACKS 0.996^47^. Low quality (Phred score below 10 averaged on 15 bp sliding windows) and monomorphic sequences were filtered out, with a minimum coverage of three and stacking up to 3% of divergence between reads.

Custom R scripts were developed for additional filtering and SNP calling. First, loci with more than five SNPs were discarded in an effort to exclude repetitive elements, and a threshold of eight reads per genotype was applied. Second, tri-allelic genotypes, heterozygotes with a rare allele represented by less than 25% of the reads, and singleton were not considered. Third, SNPs were called as genotyped if sequenced in at least 70% of individuals. Finally, to exclude obvious outliers, SNPs with a F_IS_^48^ outside the range of − 0.2 and 0.2 were removed with *hierfstat*^49^, since extreme heterozygote deficits are not expected in a diploid species with separate sexes.

### Population-level analyses

Genetic structure and diversity from the microsatellite and RAD-seq datasets were investigated at the population level using *hierfstat*^49^ from standard population genetics parameters^50^. First, we computed observed heterozygosity (H_O_), expected heterozygosity (H_S_, also known as gene diversity), overall heterozygosity over populations (h_T_), allelic richness (a_R_), population specific F_ST_ (Beta^51^) and departure from Hardy–Weinberg (F_IS_). 95% Confidence Intervals (CI) for heterozygosities and F-statistics^48^ were estimated by bootstrapping 1000 times the variance components. Pearson correlations were used to compare estimates between marker sets, and to assess the influence of missing data on the RAD-seq estimates. To identify any outlier locus, we further investigated the relation between allele frequencies and observed heterozygosity for both marker sets by plotting H_S_ and their 95% confidence intervals given allele frequency and global heterozygote deficiency, computed as H_S_ = 2*p*(1 − *p*)(1 − f_IT_) ± 2[(2*p*(1 − *p*)(1 + f_IT_)/N]^0.5^.

Second, pairwise F_ST_ between populations^48^ were estimated and compared between marker sets by Mantel tests^52^, then used to test for isolation-by-distance (IBD^53^). Chord distances^54^ were computed (*genet.dist* function of *hierfstat*), translated in a neighbour-joining tree (*bionj* function of *ape*^55^). Furthermore, hierarchical analyses of genetic variance were carried out to test for the effects of countries and populations. To this aim, F_ST_ was partitioned between populations, within country (F_SC_), and between countries (F_CT_).

Third, we tested for sex-biased dispersal using the mAIc index implemented in *hierfstat* (*sexbias.test* function^56^), which was compared between marker sets by a Pearson correlation.

### Individual-level analyses

Genetic structure among individual genotypes was first examined by Principal Component Analyses (PCA) on each marker set, using the *indpca* function of *hierfstat*^50^.

Second, we applied the *snmf* clustering algorithm implemented in *LEA*^57^ to estimate in how many clusters the dataset can be summarized, and compute individual ancestries of each individual among these clusters. For each marker set, chains were run for K = 1–7 clusters, with 20 repetitions for each K, and blinding 10% of the data for the cross-entropy estimation (function *snmf*). Most likely Ks are reflected by low cross-entropy values. The alpha parameter was kept to the default 100 (as recommended by the authors), especially as it did not affect the results in preliminary runs. To appreciate the influence of missing data on the RAD-seq dataset, runs were also performed on a reduced dataset based only on individuals featuring above 70% of SNP matrix completeness (*n* = 172). Finally, we further explored how the number of loci influenced the retrieved patterns of genetic structure by repeating the admixture analysis on randomly selected subsets of 3,000, 2,000, 1,000, 500, 200 and 100 SNPs.

## Results

In this study, we obtained and compare genotypes from 11 microsatellite loci and 4,994 SNPs obtained by RAD-seq for the same 196 individuals sampled over 19 populations of *M. schreibersii* across seven European countries (Table 1).

### Population level analyses

**Figure 1:**
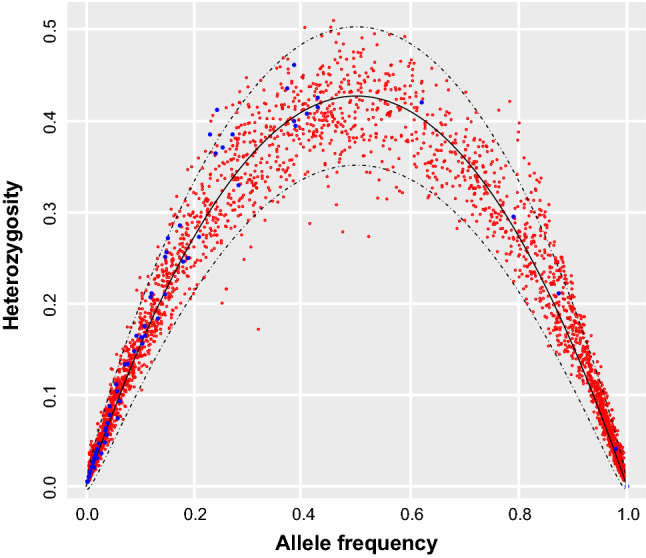
Relationship between allele frequency and observed heterozygosity for each microsatellite (blue) and SNP allele (red). The solid black line correspond to the expected heterozygosity H_S_ = 2*p*(1 − *p*)(1 − f_IT_), and the dotted lines to the 95% confidence interval of H_S_.

Population-based statistics (summarized in Table 1) showed mostly consistent patterns of diversity and structure. Microsatellite loci were more polymorphic than SNPs obtained by RAD-seq. Microsatellites bear between 3 and 18 alleles, a mean observed heterozygosity H_O_ of 0.537 (0.383–0.671), a mean expected heterozygosity H_S_ of 0.557 (0.395–0.694) and an overall heterozygosity H_T_ of 0.588 (0.418–0.734). These values are much larger than the diversity measured at bi-allelic SNPs, with a mean H_O_ of 0.122 (0.119–0.126), mean H_S_ of 0.129 (0.125–0.133) and mean H_T_ of 0.139 (0.134–0.143). Departure from Hardy Weinberg equilibrium overlapped between marker sets (SNPs F_IS_ = 0.053 [0.045–0.057]; microsatellite F_IS_ = 0.035 [0.011–0.058]). Most SNPs and microsatellite alleles show good concordance between observed and expected heterozygosities (Fig. 1). For the RAD dataset, H_O_ and F_IS_ were sensitive to the completeness of the SNP genotype matrix, as reflected by lower heterozygosity and higher F_IS_ in populations with higher proportions of missing data (Supplementary File S2). Nevertheless, the effect was mainly influenced by a single population that featured nearly 50% of missing data (loc. 9, France), and was not significant for H_S_ (Supplementary File S2).

**Figure 2:**
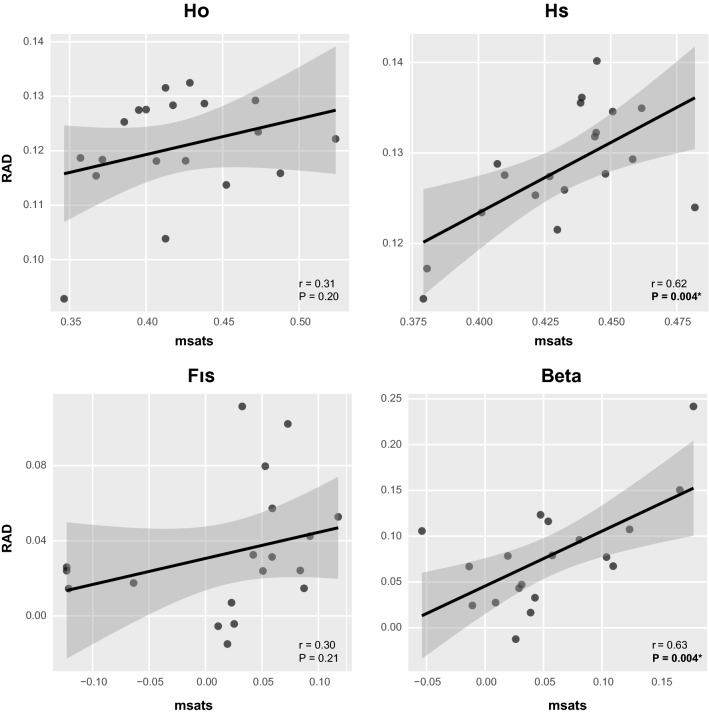
Correlation between population genetic estimates measured from microsatellites and SNPs obtained from RAD-seq. H_O_: observed heterozygosity; H_S_: expected heterozygosity; F_IS_: inbreeding coefficient; Beta: population specific F_ST_. Pearson’s correlation coefficients (r) and associated *P* values are displayed.

Microsatellite and RAD-seq estimates were significantly correlated for H_S_ (r = 0.62; *P* = 0.004) and population specific F_ST_ (r = 0.63; *P* = 0.004) (Fig. 2). The correlation was also large for a_r_ (r = 0.68; *P* = 0.001), as expected since a_r_ co-varies with h_s_. In contrast, the relationships were not significant for f_is_ and h_o_ (Fig. 2).

**Figure 3:**
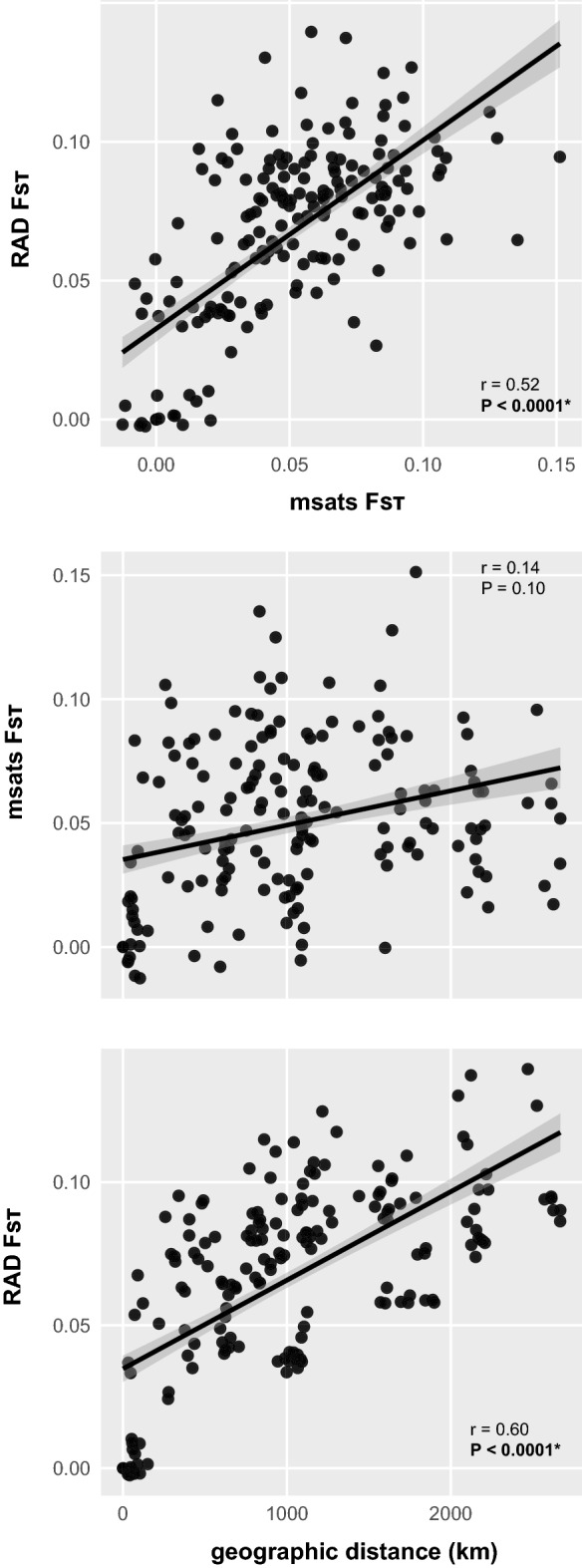
Correlation between pairwise F_ST_ computed from RAD-seq SNPs and microsatellites, and with geographic distances. Coefficient of Mantel tests (r) and their associated *P* values are displayed.

**Figure 4:**
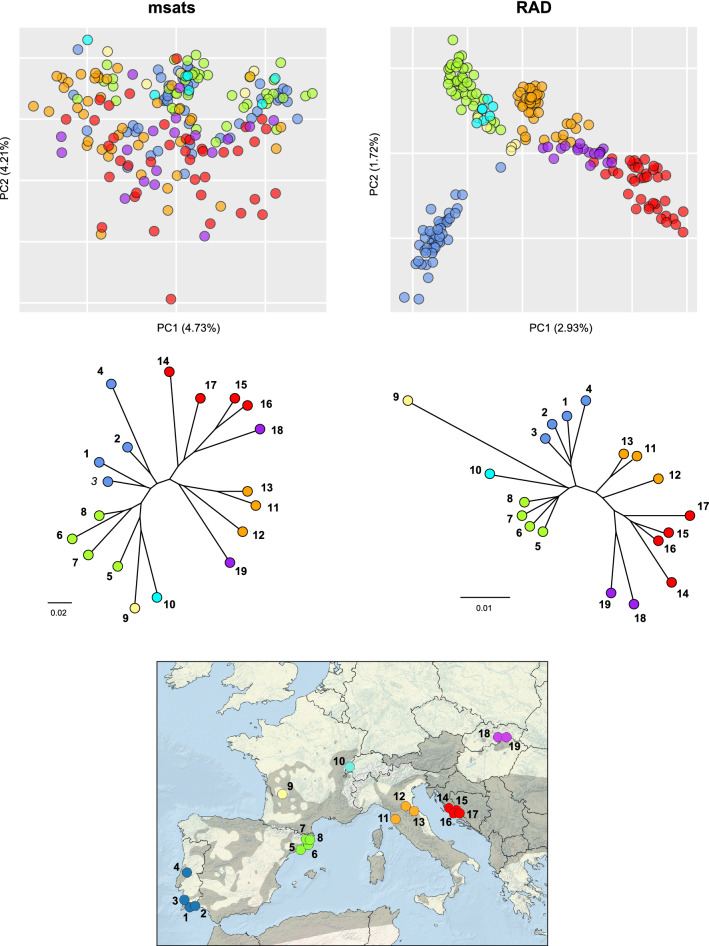
(**A**) Principal Component Analysis (PCA) of individual genotypes and (**B**) Neighbor-joining tree of Chord distances, as inferred from microsatellites and RAD-seq SNPs. The bottom map shows sampling locations (numbers as in Table 1) with color codes to distinguish populations by countries. The grey background shows the distribution of the species, according to^41^.

Pairwise population differentiation (f_st_) was stronger when estimated from SNPs (mean F_ST_ = 0.068 [0.065–0.071]) than from microsatellites (mean F_ST_ = 0.053 [0.041–0.066]). There was a significant correlation between microsatellite- and SNPs-based F_ST_ estimates (Mantel test, Fig. 3), but a significant pattern of isolation-by-distance was recovered only from the SNP dataset (Mantel tests, Fig. 3). Furthermore, both sets of f_st_ produced similar trees based on Chord distances, where populations are mostly grouped by countries (Fig. 4).

Accordingly, analyses of genetic variance revealed stronger differentiation between countries (f_ct_) than between sub-populations within countries (f_sc_), especially when estimated from SNPs: for microsatellites, f_sc_ was 0.023 (0.013–0.034) and f_ct_ was 0.035 (0.026–0.043), whereas for SNPs, f_sc_ was 0.019 (0.017–0.020) and f_ct_ was 0.058 (0.056–0.061).

Both datasets showed no evidence for sex-biased dispersal (microsatellites: t = − 0.91, *P* = 0.37; SNPs: t = − 0.62, *P* = 0.53). The corrected mAIc for microsatellites and SNPs are strongly correlated (r = 0.49, *P* < 0.001).

### Individual-level analyses

In contrast to the mostly congruent population-level analyses, the SNPs dataset provided a much higher resolution than microsatellites in individual-based analyses.

The microsatellite PCA showed no clear patterns of structure, as individuals of various origins overlap on the first axes. In contrast, axes 1 and 2 of the PCA built from SNP genotypes sorted most individuals by their country of origin (Fig. 4).

**Figure 5:**
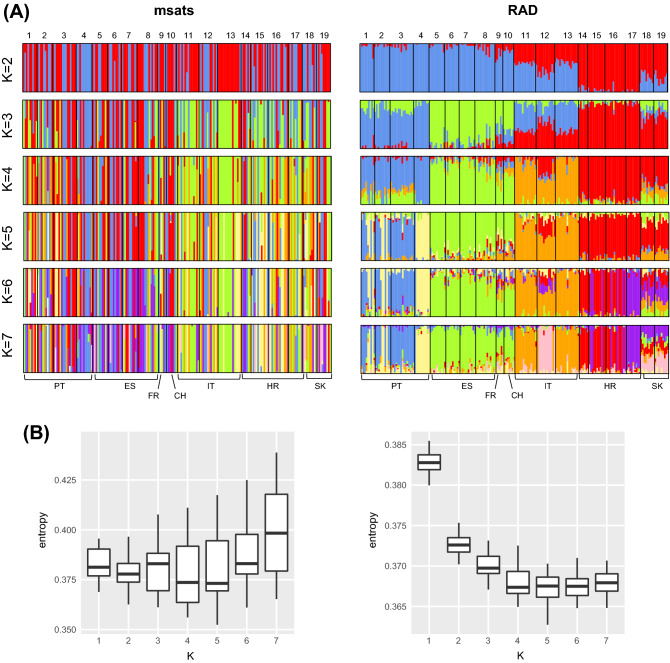
Admixture analyses on the microsatellite (left) and the RAD-seq SNPs data (right). (**A**) Individual ancestry coefficients for K = 2–7. (**B**) Cross-entropy boxplot for each K.

Accordingly, clustering analyses by the *snmf* algorithm showed radically different patterns of genetic structure (Fig. 5). With microsatellites, cross-entropy hardly highlighted a particular number of clusters. With SNPs, it clearly shows that at least four clusters are needed to explain the structure present in the dataset. Like the PCAs, the microsatellite ancestry coefficients did not convey a meaningful geographic pattern while individuals were assigned according to their populations of origin and country based on SNPs (Fig. 5).

**Figure 6:**
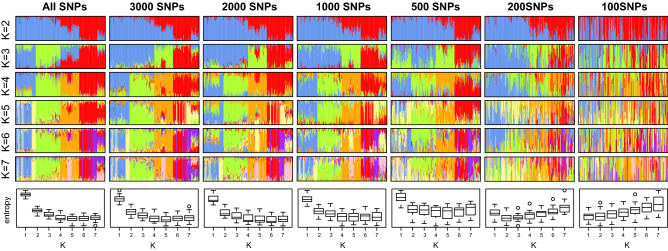
Admixture analyses on subsets of SNP data. Global patterns of structure (four main clusters in blue, green, orange and red) tend to vanish below 500SNPs, and 1000SNPs are needed to properly recover the regional substructure (yellow, purple and pink clusters).

Admixture analyses conducted on subsets of SNPs recovered the “true” signal of genetic structure (as obtained from the full dataset), depending on the number of retained markers and the complexity of the clustering scheme (number of K groups) (Fig. 6). With large datasets (≥ 500–1000 SNPs), broadly similar results are obtained, even for estimating ancestries among many groups. The picture becomes blurry with only 200 SNPs (especially for higher values of K), and cross-entropy suggests that two groups mostly exist in the dataset. Finally, the analysis with only 100 SNPs was essentially uninformative.

### Geographic patterns

**Figure 7:**
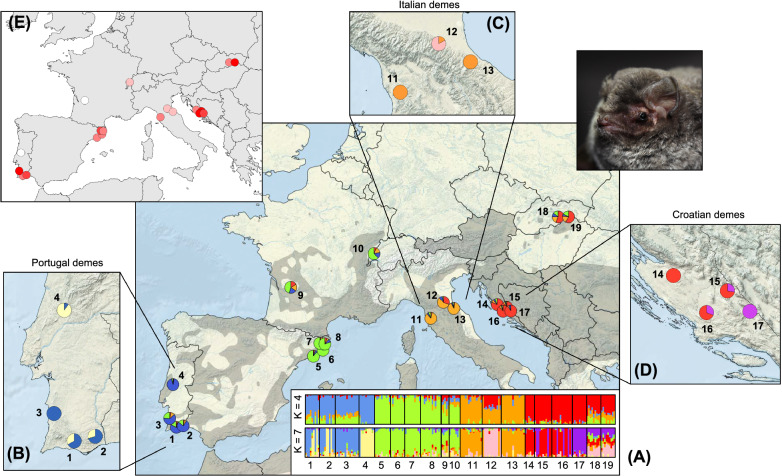
Overview of the genetic structure and diversity of *M. schreibersii* in Europe, as inferred from the SNP dataset. (**A**) Clustering analyses based on the preferred *snmf* solution of four groups (main map and upper barplots) and based on the seven groups solution, which identify distinct demes within several regions (lower barplots). (**B**–**D**) Within-populations proportions of individuals predominantly assigned to either regional deme. (**E**) Geographic variation of h_S_, from 0.11 (white) to 0.14 (red). On the main map, the grey background shows the distribution of the species, according to^40^. Photo credit: PC.

Despite the overall low genetic differentiation (see above), the SNPs dataset recovered clear patterns of genetic structure across the sampled range of *M. schreibersii* (Figs. 4, 5, 6, 7). Specifically, the four main clusters identified by the preferred *snmf* analysis roughly distinguished bats from Portugal (loc. 1–4; blue in Fig. 7); northwestern Spain (loc. 5–8; green in Fig. 7); Italy (loc. 11–13; orange in Fig. 7); and Croatia (loc. 14–17; red in Fig. 7). Individuals from the other populations showed ancestries from several clusters (Fig. 7), and were accordingly placed at intermediate positions on the PCA’s first two axes (Fig. 4): those from France (loc. 9) and Switzerland (loc. 10) are mostly “Spanish”, with substantial contributions from all other clusters; individuals from one Italian population (loc. 12) showed equal contributions from the “Italian” and the “Croatian” clusters; individuals from the two Slovakian populations are mostly “Croatian”, with substantial contributions from the “Italian” and “Spanish” cluster. Separate analyses limited to 172 individuals with less than 30% of missing data retrieved nearly identical results (Supplementary File S3).

Detailed inspections of the admixture runs with up to seven groups revealed remarkable substructure among the main Portuguese, Italian and Croatian clusters that allow tracking individual movements at the regional level. In Portugal, bats caught at loc. 3 and 4 differ genetically (blue and yellow clusters in Fig. 7), and pure individuals of both sub-clusters were found in syntopy at loc. 1 and 2. In Italy, loc. 12 individuals were identified as a different sub-cluster (pink in Fig. 7), except for two that seemingly originated from the main Italian group found in loc. 11 and 13 (orange in Fig. 7). In Croatia, loc. 17 represents a distinct population (purple in Fig. 7), but loc. 14–16 also host some of its individuals. These patterns are confirmed by inspection of additional PCA axes (Supplementary File S4).

Genetic diversity (H_S_, which was not influenced by missing data), varied greatly among populations, being generally higher in Portugal, northwestern Spain and Croatia (Fig. 7).

## Discussion

Flying vertebrates are expected to feature shallow genetic differentiation between populations due to their high movement capabilities^58,59^, but at the same time, complex patterns of regional substructure may arise typically because of philopatric and migratory behaviors^31^, combined with the biogeographic history of populations. With respect to these aspects, here we aimed to estimate genetic structure in the mobile, migratory and putatively philopatric bat *M. schreibersii* across Europe, comparing approaches based on few highly polymorphic loci (microsatellites) *vs* many bi-allelic loci (SNPs obtained by RAD-seq).

### Little yet genuine phylogeographic structure in *M. schreibersii*

Both sets of markers recovered low f_ST_ estimates across the European range of *M. schreibersii*, namely 0.05 (microsatellites) or 0.07 (SNPs) on average. This is in accordance with previous microsatellite genotyping that found little genetic differentiation across the range of the species^40^, as well as the fact that a single mitochondrial lineage inhabits Europe^39^. As these studies concluded, the lack of phylogenetic divergence between populations separated by thousands of kilometers, and the marked isolation-by-distance, suggest high connectivity.

Unlike previous studies, however, we did recover genetic structure across Europe with genome-wide SNPs. We also found similar levels of diversity between populations, especially between the eastern- and westernmost ones (Portugal and Croatia). Both results are somewhat surprising if one assumes that the species post-glacially expanded across Europe from a single Anatolian refugium—the currently accepted biogeographic scenario, based on higher mitochondrial and microsatellite diversity in Anatolia^39,40^. Under this scenario, a single genetic cluster and a westward longitudinal gradient of genetic variation would have been expected across the whole continent (i.e., lower diversity in Iberia compared to the Balkans). Whether the glacial ranges of *M. schreibersii* also encompassed Mediterranean Europe, in addition to Anatolia, thus remains an open question. In fact, the structure we identified in Europe would even be consistent with distinct historical refugia across the Iberian, Apennine and Balkan Peninsula, perhaps subdivided into micro-refugia (e.g., Iberia). In this respect, the puzzling ancestry of Slovakian bats could reflect recent admixture between the Croatian group and a gene pool that we have not sampled i.e., from another separate refugium, for instance, Anatolia. The last lines on the biogeography of *M. schreibersii* are thus yet to be written. A genomic survey encompassing both European and extra-European populations to thoroughly infer range-wide patterns of genetic diversity, structure, and admixture, perhaps combined with environmental niche reconstruction to track habitats suitable for the species during the last glacial maximum, will hold key to disentangle among the one refugium *vs* several refugia hypotheses.

Suitable glacial habitats were modelled in southern Europe for other species of bats with roughly similar distributions, e.g., *Nyctalus leisleri*^60^ or *Plecotus austriacus*^61^. Like *M. schreibersii*, European bats in general show little phylogeographic structure^60,62,63^, or sometimes recent geographic isolation resulting from the last glacial stage, as in *Rhinolophus ferrumequinum*^64^ and *Rhinolophus hipposideros*^65^. Yet, the lack of clear genetic structure between candidate refugial regions does not necessarily imply a single refugium scenario, as here previously assumed for *M. schreibersii*^39,40^, as well as in other species, e.g., *Myotis bechsteinii*^66^. In mobile organisms, genetic signals for multiple refugia (e.g., several glacial lineages) can quickly disappear in the event of gene flow and lineage fusion^67^, and become undetectable with few bi-parentally inherited microsatellite loci. Long-lasting signals may persist in the mitochondrion, but only if females are more philopatric than males. In a similar fashion, multiple refugia exist in the European Barn Owl (*Tyto alba*)—a fast-dispersing bird with a similar circum-Mediterranean distribution—but it was likewise only retrieved from genomic data, namely whole-genome sequencing^59^, and not microsatellites^68^. Classic biogeographic paradigms characteristic of terrestrial vertebrates, such as the long-term persistence in separate Mediterranean refugia^69,70^ and their regional differentiation in refugia-within-refugia^71^ might thus apply to bats and birds even if microsatellite studies have so far suggested otherwise. That said, some widespread Holarctic bat species may truly be genetically homogenous. For instance, the north-American *Myotis lucifugus* lacks population differentiation, as measured from both conventional (microsatellites + mtDNA^72^) and genomic markers (RAD-seq^73^; low coverage genome^74^).

### Regional genetic structure by social organization

Despite the relatively weak range-wide differentiation, the high-resolution offered by our RAD-seq dataset recovered regional signals of population sub-structuring, which can be interpreted in the light of the social habits of *M. schreibersii*. In bats, mating and breeding colonies are often spatially separated^37,75^, and their average genetic diversity and relatedness vary accordingly^66^. In addition, females are usually the philopatric sex, and males the dispersing sex, which should affect mitochondrial *vs* nuclear patterns of genetic structure^31^.

In *M. schreibersii*, ringing data have suggested that both males and females were philopatric^36^, and we also recovered no evidence for sex-biased dispersal. Male-biased dispersal was however detected by a regional population genetic study focusing on the Portuguese colonies^38^. The discrepancy probably lies in the larger geographic scale of our study compared to the latter: sex-biased dispersal can only be retrieved if the dataset contains a fair amount of dispersers^56^, which are obviously in lower proportions at the continental compared to the regional scale, especially if dispersal remains rare overall.

Furthermore, we detected syntopic individuals with unrelated ancestries in several nursing sites of Portugal (loc. 1–2), Italy (loc. 12), and Croatia (loc. 15–16), yet with little signs of local admixture—which would have been flagged by intermediate ancestry coefficients. These sites thus act as “regional hubs” where individuals of various origins temporarily meet yet remain genetically differentiated. Such observations are consistent with the idea that *M. schreibersii* is a keen seasonal migrator (moving a lot between colonies), but a weak disperser (exchanging little genes between them)^36^. Hence, the social organization of the species momentarily shapes the genetic diversity of its populations, which may differ between nursing, mating and hibernating sites. The factors triggering genome-wide divergence between co-occurring nursing individuals remain elusive, and may include isolation-by-distance, assortative mating, or a meta-population structure—which has been documented in bats^76,77^. More insights will come from assessing fine-scale geographic patterns of genetic diversity from the mating sites i.e., the “true” origins of individuals, and test whether these are genetically isolated.

### Inferring genetic structure when there is little

In recent years, several studies have compared traditional genetics *vs* genomics methods to infer population diversity and structure, especially in the context of conservation^78–81^. In their synthesis, Sunde et al.^30^ concluded that RAD-seq performed as well or better than microsatellites, as similar trends of clustering and heterozygosity were always recovered from both types of markers (see their Table 1). However, most previous work was biased towards aquatic organisms (especially fish), with relatively pronounced genetic structures reflected by higher pairwise f_ST_ e.g., > 0.1 in^30,81^ and 0.4 on average in^78^.

Unlike previous comparative studies, here the generally low genetic differentiation among European populations of *M. schreibersii* offers a framework to evaluate the performance of genotyping methods to recover shallow patterns of genetic structure. In this respect, microsatellites were out-performed by SNPs obtained from RAD-seq in most analyses. While the difference in population-based F-statistics was not so obvious (Figs. 1, 2, 3, 4), individual-based analyses (PCA, *snmf* clustering) were much clearer when inferred from SNPs (Figs. 4, 5). This result is somewhat expected given the weak divergence among our populations, which may have maintained high effective sizes in large (although potentially composite) refugia (see above). In such cases, drift does not counteract the effect of mutation at fast-evolving microsatellites, which results in high yet shared diversity (homoplasy) between regions, hence uninformative of population structure. By capturing less diversity at multiple, putatively more conserved loci, SNPs clearly provided a more reliable picture of the entire genome: not only could they recover the subtle patterns of range-wide phylogeographic differentiation, but they also informed on local population structure. Moreover, because estimates of allele frequencies based on a few individuals will have higher variance for multi-allelic compared to bi-allelic markers, genomic SNPs may be particularly more informative than microsatellites when samples sizes are modest. Microsatellite analyses may require up to 25–30 individuals per population for accurate population genetic inferences^82^, which is often unrealistic due to biological and field constraints (e.g., 5–15 samples per population here). Therefore, our study extends the conclusions of Sunde et al.^30^ that SNPs provide a significant upgrade to microsatellite-based population genetics, even more so in mobile organisms with shallow genetic structure, as well as for tracking individual movements.

Going beyond the molecular approach itself, can we estimate what numbers of genomic loci are needed to reliably infer population differentiation in weakly structured organisms? In a study on red mangroves, Hodel et al.^79^ reported gradual improvements with different filtering stringencies that increased their datasets from ~ 200 to ~ 25′000 SNPs (but also increased missing data from 11 to 78%). Our sub-setting analyses largely agrees with their conclusions. While our 100–200 SNPs datasets were mostly uninformative, 500 SNPs (10 times less than our initial dataset) still recovered most of the shallow structure of *M. schreibersii*, and at least 1,000 SNPs were required to investigate the regional substructure (Fig. 6). Hence, depending on the biological question and organisms, meaningful patterns might be recovered from lesser sequencing efforts (i.e., retrieving fewer SNPs) or genetically impoverished species (i.e., containing fewer SNPs to begin with). Special precautions should nevertheless be taken to acknowledge missing data, especially when inferring population genetic estimates based on heterozygosity. For a given coverage threshold, heterozygous sites are typically discarded more often than homozygous sites by bioinformatics filters, because fewer reads per allele are sequenced. In turn, heterozygous SNPs are more difficult to retain than homozygote SNPs in low-quality samples, which contributes to artificially deplete H_O_ and inflate F_IS_. While this issue can be circumvented by exploring the filtering parameters, an alternative is to consider genetic diversity based on allelic richness and H_E_, which both have the advantage to rely on the number of alleles per population rather than the number of heterozygotes.

### Conservation implications

Our study provides applied lessons for the conservation of *M. schreibersii*, which has sustained massive die-offs in Spain, France and Portugal in 2002, associated to the emergence of a new filovirus, the Lloviu virus (LLOV)^83,84^. As previously recommended^36^, the genetic differentiation documented at the regional scale calls for protecting all the sites used by the species, both to ensure regional connectivity between the hubs visited by seasonal migrants and to preserve the genetic uniqueness of the different demes, as it might convey components of local adaptation. Moreover, some of the most isolated sites in Portugal (loc. 4) and France (loc. 9) show the lowest genetic variation, which may reflect genetic bottlenecks ensuing the population collapses from the 2000s^83^. These populations should thus remain under constant care, especially if their eroded genetic diversity negatively affect fitness^85^. Besides, recovery programs have proven very efficient in *M. schreibersii*^86^. Finally, the genetic groups and individual movements identified inform on the risk assessment of future disease outbreaks, in the context of the possible re-emergence of the Lloviu virus, which has been re-detected since 2016 in an Hungarian colony^87^ and was shown potential to infect human cells ^88^. The spreading may be fast within regions—where bat populations are either panmictic, or in close contact during nursing—but could be slower at the continental scale given the structuration between European countries.

Weak genetic differentiation at the global scale despite rare dispersal and marked structure at the local scale may appear counter-intuitive, yet such pattern is not unheard of among geographically-scattered species. An analogous situation was documented in the common hippopotamus, which expanded across Africa in a single wave (homogenizing its rich genetic diversity), before contemporary habitat fragmentation created barriers to dispersal and drove genetic bottlenecks^89^. In a similar fashion, the ongoing fragmentation of *M. schreibersii*’s range could have contributed to promote the genetic differentiation between the Portuguese, Spanish, French, Swiss and Croatian populations. We argue that the lack of resolution offered by traditional population genetics potentially hamper the identification of subtle patterns of genetic structure in phylogeographically shallow taxa, and that the situation of the declining *M. schreibersii* might be more common that previously assumed.

## Supplementary Information

Supplementary Information.

## Data Availability

Microsatellite and SNP matrices are available at the following link: https://doi.org/10.5061/dryad.4xgxd25d2. R scripts are available upon demand to Jérôme Goudet (jerome.goudet@unil.ch) and Christophe Dufresnes (christophe.dufresnes@hotmail.fr).
