# Supplementary material for: Inferring genetic structure when there is little: population genetics versus genomics of the threatened bat Miniopterus schreibersii across Europe
Source: Sci Rep. 2023 Jan 27;13:1523. doi: 10.1038/s41598-023-27988-4 (PMC9883447; doi:10.1038/s41598-023-27988-4)

Supplementary Material for:

Inferring genetic structure when there is little: Population genetics vs genomics of the threatened bat *Miniopterus schreibersii* across Europe

Christophe Dufresnes, Ludovic Dutoit, Alan Brelsford, Fardo Goldstein-Witsenburg, Laura Clément, Adria López-Baucells, Jorge Palmeirim, Igor Pavlinić, Dino Scaravelli, Martin Ševčík, Philippe Christe, Jérôme Goudet

Content **page**

File S1: ddRAD-seq protocol 2
File S2: Missing data and population genetics parameters 4
File S3: Missing data and clustering analyses 5
File S4: PCA on the RAD-seq SNP data up to axis 8 6

File S1: ddRAD-seq protocol

We carried out genotyping-by-sequencing using a procedure based on [45], with modifications to obtain a lower marker density. DNA was first digested by two HF restriction endonucleases, *Eco*RI and *Sac*I (NEB inc.).

Precipitated DNA samples (60-120ng) in 0.1X TE (pH 8) were digested with 5 units (U) of *Eco*RI and 2U of SacI endonucleases in a 9µl reaction completed of 1µl 10x T4 Buffer, 0.52µl 1M NaCl and 0.52µl 1mg/ml BSA. Digestion was performed at 37°C for 8 hours. Endonucleases were inactivated by heating up samples at 65°C for 20 minutes.

Restricted fragments were then processed through a ligation. Adaptors were built of two single-stranded oligos. Each one contained an Illumina adaptor for binding of the sequencing (primer, an individual barcode of 7bp, the complementary bases to the cut site and a supplementary base that prevent removing of the adaptor during the ligation.

Oligo 1:
*Eco*RI side: 5'-CTCTTTCCCTACACGACGCTCTTCCGATCT + 7 bp individual barcode+C-3';
*Sac*I side: 5'-AGATCGGAAGAGCACACGTCTGAACTCCAGTCA -3'

Oligo 2:
*Eco*RI side: 5'-AATTG+ 7bp individual barcode + AGATCGGAAGAGCGTCGTGTAGGGAAAGAGTGT-3' 152
*Sac*I side: 5'-CTGGAGTTCAGACGTGTGCTCTTCCGATCTAGCT-3'

There is no barcode on the *Sac*I side because sequencing only occurs from the *Eco*RI side. Adaptors were annealed by mixing 1µM of a pair, heated up the mix to 95°C and slowly cooled down to room temperature to obtain one double stranded adaptor. The ligation reaction consisted of 67U of T4 DNA ligase (NEB inc.), 0.13µl 1M NaCl , 0.13µl mg/ml BSA, 0.16µl 158 10X T4 Buffer and 1µl *Sac*I adaptor added to the digestion product for a total volume of 159 11.6µl maintained at 16°C for 6 hours. Ligation products were then diluted to 200µl with 0.1 160 X TE Buffer.

Fragments were then PCR amplified using 4 different Illumina primers matching the adaptors:

ILLPCR1:
A*A*TGATACGGCGACCACCGAGATCTACACTCTTTCCCTACACGACGCTCTTCCGATCT
ILLPCR2-bar04:
C*A*AGCAGAAGACGGCATACGAGATTGGTCAGTGACTGGAGTTCAGACGTGTGCTCTTCCGATCT
ILLPCR2-bar06:
C*A*AGCAGAAGACGGCATACGAGATATTGGCGTGACTGGAGTTCAGACGTGTGCTCTTCCGATCT

ILLPCR2-Bar12:
C*A*AGCAGAAGACGGCATACGAGATTACAAGGTGACTGGAGTTCAGACGTGTGC
TCTTCCGATCT)*

Each ILLPCR2 primer incorporates a unique 6bp index into the *Sac*I end of restriction fragments. ILLPCR1 has been combined with each of the 3 different ILLPCR2 primers to create 3 multiplexed libraries with up to 96 individuals (i.e.number of 7bp barcodes available). This combination of individual barcodes and Illumina primers allowed us to increase the number of individuals sequenced without the purchase of additional expensive individual barcodes. Two different PCR reactions were performed for each sample to control for errors (30 cycles; 98°C for 30s; 30 cycles of: 98°C for 20s, 60°C for 30s, 72°C for 40s; final extension at 72°C 183 for 10 min). Each 20µl PCR reaction consisted of 0.2U of Phusion Hot Start high quality 184 DNA Polymerase (NEB inc.), 4µl diluted ligation product, 0.16µl 25µM dNTP, 4µl 5X 185 Phusion HF Buffer, 0.4µl 50µM MgCl2, 0.15µl 100% DMSO and 0.134µl of PCR primer at 186 5µM each. A final cycle of 98° C for 3 min, 60° C for 2 min, 72° C for 12 min was run after 187 adding 2µl to the amplified samples (0.4µl 5X Phusion HF Buffer, 0.134µl primer mix and 188 0.16µl 25µM dNTP).

SPCR samples were partially evaporated on the bench to reduce the volume to approximately 10µl and then all PCR products were pooled together. From the pooled stock, 720µl of pooled PCR products (18 x 40µl pooled stock + 9µl loading buffer in each well) were run on a 2.5% 193 agarose gel for 2 hours and 30 minutes at 100V. Gels were then excised between 300-500p 194 using the upper part of a 1000µl pipette tip. UV exposure times were minimized to limit DNA degradation. Agarose pieces were purified using Min Elute Gel Purification Kit (Qiagen). Finally, a Chloropane precipitation (see supplementary material) was run to concentrate gel extraction products and purify DNA. The libraries were quantified using a Qubit® Fluorometer and fragment length distribution was checked using a Bioanalyzer (Agilent).)) The 3 libraries were sequenced on 2 lanes of Illumina HiSeq 2000 (Lausanne Genomic Technologies Facility, Switzerland).

File S2: Relationship between the proportion of missing data in the SNP dataset and the retrieved population genetic indices. H_O: observed heterozygosity; H_S: expected heterozygosity; F_{IS}: inbreeding coefficient; Beta: within-population F_{ST}. Pearson's correlation coefficients (r) and associated p-values are displayed.

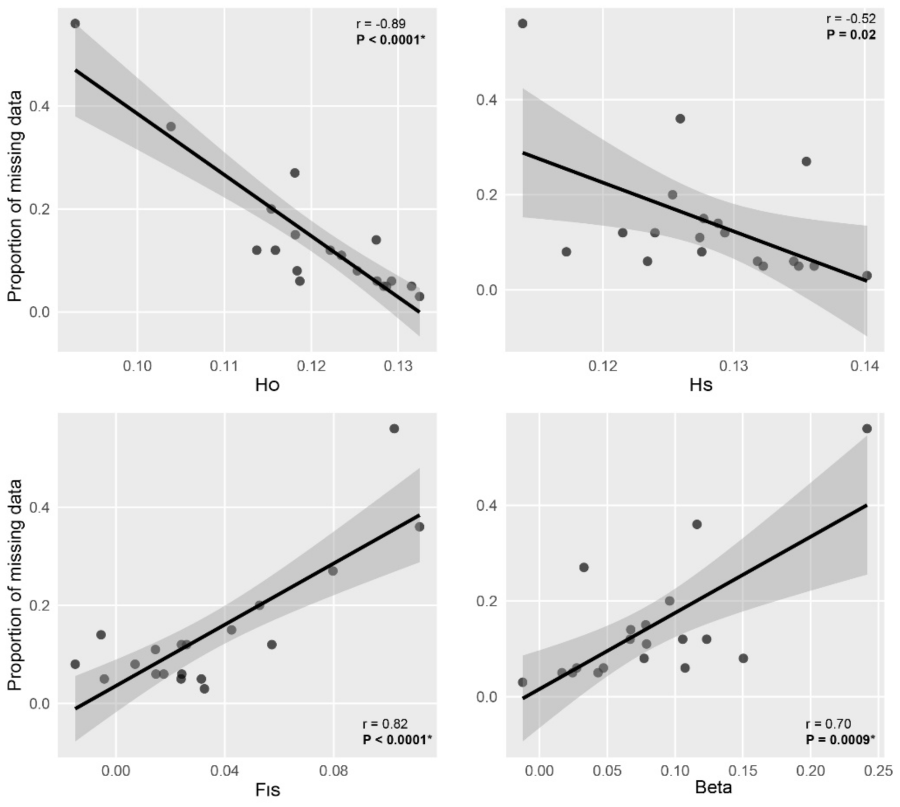

File S3: Clustering analyses on the RAD-seq SNPs data limited to the 172 individuals that feature no more than 30% of missing data. Results are nearly identical to those inferred from the full dataset (Fig. 5).

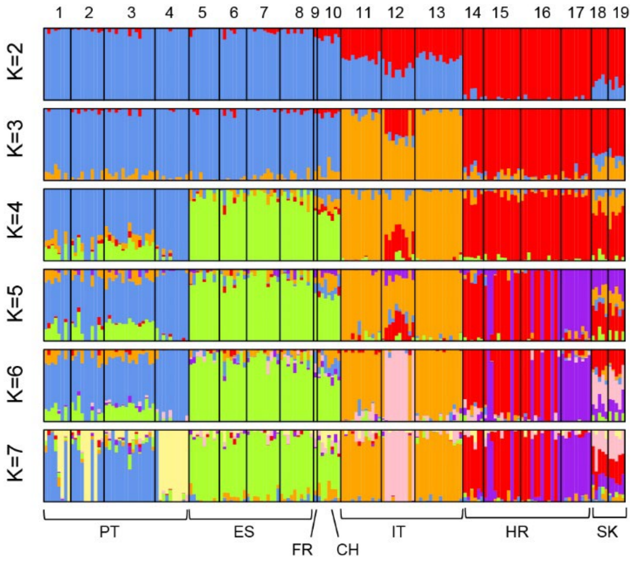

File S4: PCA on the RAD-seq SNP data up to axis 8. Colors follow Fig. 5. The substructure detected within countries (Fig. 5) by the *snmf* analysis is also retrieved, namely in Croatia by axis 4, in Portugal by axes 5, 6 and 8, and in Italy by axis 7.

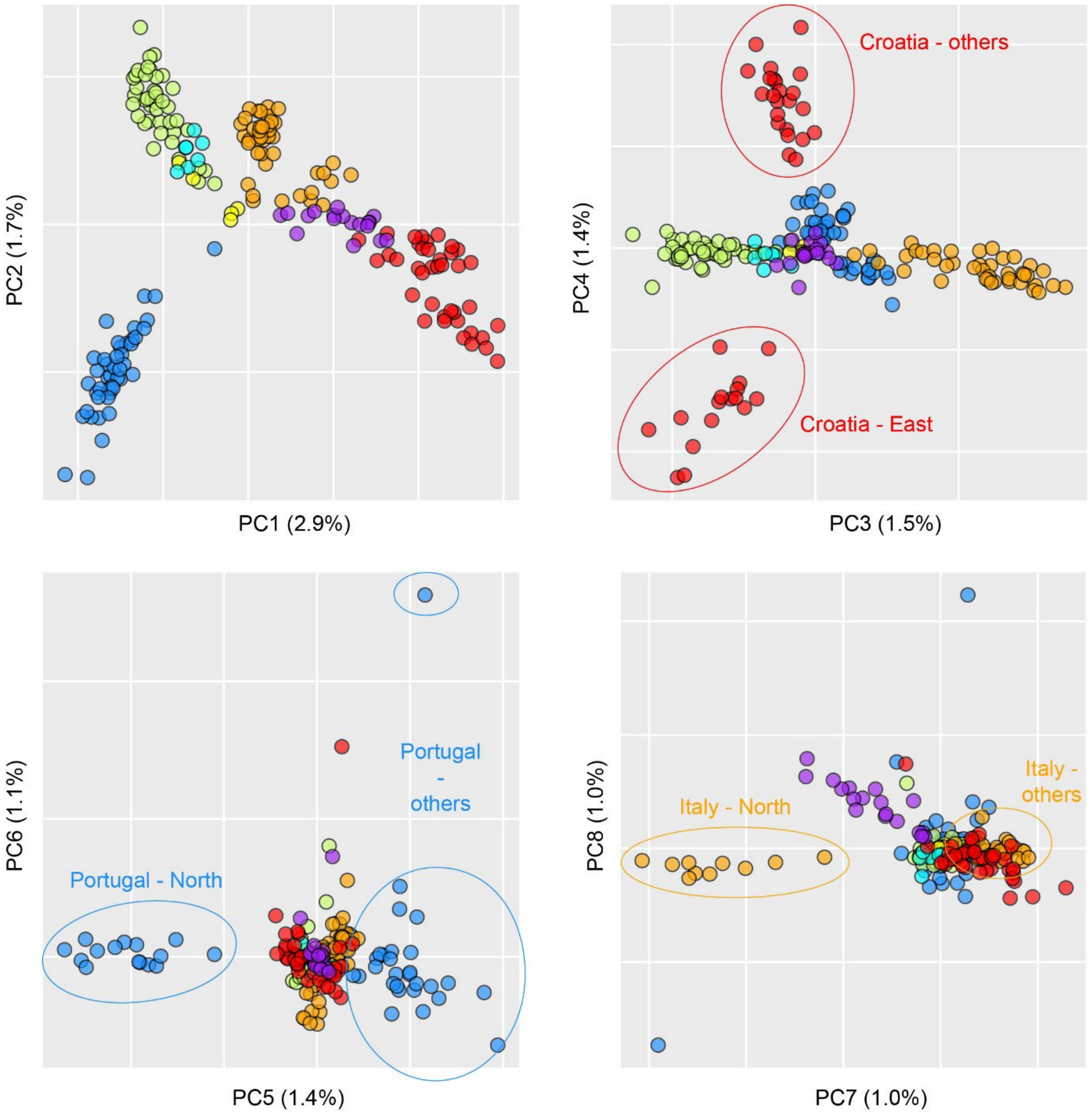

Supplement: Supplementary file 1 — Supplementary Information. [file 41598_2023_27988_MOESM1_ESM.pdf]
